# Multi-Channel Morphological Profiles for Classification of Hyperspectral Images Using Support Vector Machines

**DOI:** 10.3390/s90100196

**Published:** 2009-01-08

**Authors:** Javier Plaza, Antonio J. Plaza, Cristina Barra

**Affiliations:** Department of Technology of Computers and Communications, University of Extremadura / Escuela Politécnica de Cáceres, Avenida de la Universidad s/n, E-10071 Cáceres, Spain; E-Mails: jplaza@unex.es; crbaar@unex.es

**Keywords:** Hyperspectral imaging, remote sensing, morphological profiles, spatial-spectral classification, vector ordering, land-cover classification, support vector machine (SVM)

## Abstract

Hyperspectral imaging is a new remote sensing technique that generates hundreds of images, corresponding to different wavelength channels, for the same area on the surface of the Earth. Supervised classification of hyperspectral image data sets is a challenging problem due to the limited availability of training samples (which are very difficult and costly to obtain in practice) and the extremely high dimensionality of the data. In this paper, we explore the use of multi-channel morphological profiles for feature extraction prior to classification of remotely sensed hyperspectral data sets using support vector machines (SVMs). In order to introduce multi-channel morphological transformations, which rely on ordering of pixel vectors in multidimensional space, several vector ordering strategies are investigated. A reduced implementation which builds the multi-channel morphological profile based on the first components resulting from a dimensional reduction transformation applied to the input data is also proposed. Our experimental results, conducted using three representative hyperspectral data sets collected by NASA's Airborne Visible-Infrared Imaging Spectrometer (AVIRIS) sensor and the German Digital Airborne Imaging Spectrometer (DAIS 7915), reveal that multi-channel morphological profiles can improve single-channel morphological profiles in the task of extracting relevant features for classification of hyperspectral data using small training sets.

## 1. Introduction

**Figure 1.**
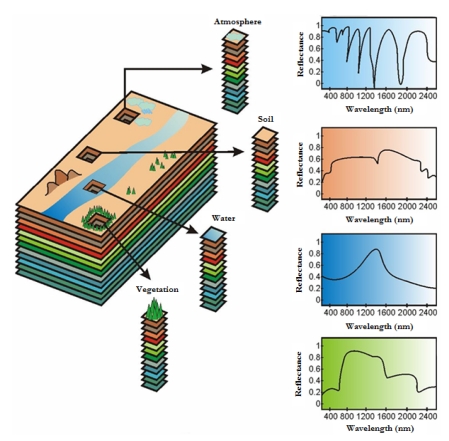
The concept of hyperspectral imaging illustrated using NASA's AVIRIS sensor.

Hyperspectral imaging (also known as imaging spectroscopy) is an emerging technique that has gained tremendous popularity in many research areas, most notably, in remotely sensed satellite imaging and aerial reconnaissance [1]. This technique is concerned with the measurement, analysis and interpretation of spectra acquired from a given scene (or specific object) at a short, medium or long distance by an airborne or satellite sensor. Recent advances in sensor technology have led to the development of advanced hyperspectral instruments capable of collecting high-dimensional image data, using hundreds of contiguous spectral channels, over the same area on the surface of the Earth. The concept of hyperspectral imaging is linked to one of NASA's premier instruments for Earth exploration, the Jet Propulsion Laboratory's Airborne Visible-Infrared Imaging Spectrometer (AVIRIS) system [2]. As shown by Figure 1, this imager measures reflected radiation in the wavelength region from 0.4 to 2.5 μm using 224 spectral channels, at nominal spectral resolution of 10 nm. The result is an “image cube” in which each pixel is given by a vector of values that can be interpreted as a representative spectral signature for each observed material [3]. The wealth of spectral information provided by latest-generation hyperspectral sensors has opened ground-breaking perspectives in many applications [4], including environmental modeling and assessment, target detection for military and defense/security deployment, urban planning and management studies, risk/hazard prevention and response including wild-land fire tracking, biological threat detection, monitoring of oil spills and other types of chemical contamination.

The special characteristics of hyperspectral data sets pose different processing problems, which must be necessarily tackled under specific mathematical formalisms. For instance, several machine learning techniques have been applied to extract relevant information from hyperspectral data sets [5]. Due to the small number of training samples and the high number of features generally available in hyperspectral imaging applications, reliable estimation of statistical class parameters is a challenging goal. As a result, with a limited training set, classification accuracy tends to decrease as the number of features increases (this is known as the Hughes effect [3]). Another challenge in hyperspectral image analysis is the fact that each spectral signature generally measures the response of multiple underlying materials at each site. For instance, the pixel vector labeled as “vegetation” in Figure 1 may actually be a mixed pixel comprising a mixture of vegetation and soil, or different types of soil and vegetation canopies. Mixed pixels exist for one of two reasons [4]. Firstly, if the spatial resolution of the sensor is not high enough to separate different materials, these can jointly occupy a single pixel, and the resulting spectral measurement will be a composite of the individual spectra. Secondly, mixed pixels can also result when distinct materials are combined into a homogeneous mixture (this circumstance is independent of the spatial resolution of the sensor.) As a result, a hyperspectral image is often a combination of the two situations, where a few sites in a scene are spectrally pure materials, but many others are mixtures of materials.

A possible approach in order to deal with the high-dimensional nature of hyperspectral data sets is to consider the geometrical properties rather than the statistical properties of the classes. The good classification performance demonstrated by support vector machines (SVMs) [6] using spectral signatures as input features has been improved in previous work by resorting to mathematical morphology (MM) operations [7], which are able to select the most relevant spatial features for a subsequent classification process using both spatial and spectral information. In previous work, MM operations have been used to extract information about the size, shape and the orientation of spatial structures in single-band remote sensing images [8]. In hyperspectral image processing, MM operations have been generally applied in the spatial domain of the scene [9], i.e., to each image band of the original scene or to the first few bands resulting from a transformed version of the original hyperspectral scene using techniques such as principal component analysis (PCA) [10] or the minimum noise fraction (MNF) [11]. Variations on this idea have comprised extended morphological operations able to work on the spectral domain of the data [12-13], i.e., morphological operations applied to the entire set of bands of the original scene or to a subset of bands, in vector-based fashion. These operations were based on a standard vector ordering strategy which is revisited and extended in this work, which provides a detailed study of different vector ordering strategies and approaches for building multi-channel and mono-channel morphological profiles for hyperspectral data classification.

The remainder of the paper is organized as follows: Section 2 introduces MM and the issues involved in multidimensional ordering of feature vectors, required to extend MM operations to the spectral domain. Section 3 describes the approach followed for extension of classic MM operations to hyperspectral imagery, and provides some processing examples. Section 4 develops multi-channel morphological profiles for hyperspectral data analysis. Section 5 provides an evaluation of the proposed multi-channel morphological profiles when compared to their single-channel counterparts in the context of two different classification problems using limited training samples and an SVM classifier. Section 6 provides parallel implementations of multi-channel morphological profiles and the SVM classifier, along with performance results on two clusters of computers at NASA's Goddard Space Flight Center. Our last section concludes with some remarks and hints at plausible future research.

## 2. Classic Mathematical morphology

MM is a spatial structure analysis theory that was established by introducing fundamental operators applied to two sets [7, 14]. A set is processed by another one having a carefully selected shape and size, known as the structuring element (SE). In the context of image processing, the SE acts as a probe for extracting or suppressing specific structures of the image objects, checking that each position of the SE fits within those objects. Based on these ideas, two fundamental operators are defined in MM, namely *erosion* and *dilation*. The application of the erosion operator to an image yields an output image, which shows where the SE *fits* the objects in the image. On the other hand, the application of the dilation operator to an image produces an output image, which shows where the SE *hits* the objects in the image. All other MM operations can be expressed in terms of erosion and dilation [8]. For instance, the notion behind the *opening* operator is to dilate an eroded image in order to recover as much as possible of the eroded image. In contrast, the *closing* operator erodes a dilated image so as to recover the initial shape of image structures that have been dilated. The filtering properties of the opening and closing are based on the fact that, depending on the size and shape of the considered SE, not all structures from the original image will be recovered when these operators are applied. MM operations have found success in different application domains, including remote sensing [15].

Although MM operators were originally defined for binary images, they have been extended to gray-tone (mono-channel) images by viewing these data as an imaginary topographic relief; in this regard, the brighter the gray tone, the higher the corresponding elevation [8]. It follows that, in grayscale morphology, each 2-D gray tone image is viewed as if it were a digital elevation model (DEM). In practice, set operators directly generalize to gray-tone images. For instance, the intersection ∩ (respectively, union ∪) of two sets becomes the point-wise minimum ∧ (respectively, maximum ∨) operator [8]. In a similar way to the binary case, specific image structures are extracted/suppressed in the grayscale case according to the chosen SE. The latter is usually “flat” in the sense that it is defined in the spatial domain of the image (the x−y plane).

Extension of the concepts of binary and grayscale MM to multi-channel imagery is not straightforward. A simple approach to extend MM to multi-channel data consists in applying grayscale MM techniques to each channel separately, an approach that has been called marginal MM in the literature [16]. However, the marginal approach is unacceptable in hyperspectral imaging applications because, when MM techniques are applied independently to each image channel, there is a possibility for loss or corruption of information of the image due to the probable fact that new spectral constituents —not present in the original image— may be created as a result of processing the channels separately [8]. An alternative way to approach the problem of multi-channel MM is to treat the data at each pixel as a vector. Unfortunately, there is no unambiguous means of defining the minimum and maximum values between two vectors of more than one dimension, and thus it is important to define an appropriate arrangement of vectors in the selected vector space. In the following section, we develop a strategy for extending morphological operations to multidimensional data spaces.

## 3. Multi-Channel Mathematical morphology

### 3.1. Ordering Pixel Vectors in Hyperspectral Data

Prior to the introduction of our proposed framework for multi-channel (spectral) MM, we discuss some challenges involved in ordering of pixel vectors in hyperspectral image data. Let us consider that a hyperspectral image is denoted by *f* and defined on the N-dimensional (N-D) space, where N is the number of spectral channels or bands. Let *f*(x,y) and *f*(x′,y′) denote two pixels vectors at spatial locations (x,y) and (x′,y′), respectively, with *f*(x,y) = [*f*_1_(x,y), …, *f*_N_(x,y)]^T^ and *f*(x′,y′) = [*f*_1_(x′,y′), …, *f*_N_(x′,y′)]^T^. A traditional approach in the literature for ordering multidimensional vectors such as those resulting from hyperspectral pixels is marginal ordering (M-ordering), in which each pair of observations *f*_i_(x,y) and *f*_i_(x′,y′) would be ordered independently along each of the N channels [16]. Quite opposite, in reduced ordering (R-ordering) a scalar parameter function *g* would be computed for each pixel of the image and the ordering is performed according to the resulting scalar values. The ordered vectors would satisfy the relationship ***f***(x,y) ≤ ***f***(x′,y′) ⇒ *g*[***f***(x,y)] ≤ *g*[***f***(x′,y′)]. In this case, if the function *g* is bijective, the implication above becomes an equivalence. In partial ordering (P-ordering), the input multivariate samples would be partitioned into smaller groups, which would then be ordered. Both R-ordering and P-ordering may lead to the existence of more than one suprema (or infima) and, thus, introduce ambiguity in the resulting data. In lexicographical ordering (referred hereinafter as L-ordering), the pixel vectors would be initially ordered according to the ordered values of one of their components, e.g. the first component, *f*_1_(x,y) and *f*_1_(x′,y′). As a second step, vectors with the same value for the first component would be ordered according to the ordered values of another component, e.g. the second component, *f*_2_(x,y) and *f*_2_(x′,y′), and so on [17]. This type of ordering is not generally appropriate for hyperspectral data, where each spectral feature *as a whole* contains relevant information about the optical and physical properties of the observed land cover [2]. In addition, pixel vectors in hyperspectral imaging are usually affected by atmospheric and illumination interferers, which may introduce fluctuations in the amount of energy collected by the sensor at the different wavelength channels [4]. The incident signal is electromagnetic radiation that originates from the sun and is measured by the sensor after it has been reflected upwards by materials on the surface of the Earth. As a result, two differently illuminated pixels that belong to the same spectral constituent may be ordered inconsistently by the L-ordering and M-ordering schemes.

In this paper, we propose an application-driven vector ordering technique based on a spectral purity-based criterion, where each pixel vector in the hyperspectral image is ordered according to its spectral distance to other neighboring pixel vectors in the data. This type of ordering, which can be seen as a modification of the D-ordering available in the literature [18], has been found in previous work to be effective in capturing both spatial and spectral variability in hyperspectral data analysis [12]. An important ambiguity not sufficiently explored in previous work has to do with the fact that the ordering imposed above is not injective in general, i.e., two or more distinct vectors may output the same minimum or maximum distance. The solution suggested in [19-20] is to define an *ad hoc* total ordering for a vector space. For example, by using a space-filling curve such as a Peano curve, a total ordering is achieved since any two points on the vector space are ordered along the curve. However, the total ordering so created lacks a clear physical interpretation in the context remote sensing applications. A further approach is to apply component transformations such as the PCA or MNF and then consider the first component only [21]. This approach discards significant information that can be very useful for the discrimination of different materials. In this work, we explore the effectiveness of a more physically meaningful approach, based on exploiting all the information available in the original feature space to separate the different land cover classes in remote sensing data analysis scenarios.

### 3.2. Multi-Channel Morphological Operations

The two basic operations of mathematical morphology are erosion and dilation. Following a usual notation [8], let us consider a grayscale image *f*, defined on a space *E*. Typically, *E* is the 2-D continuous space *R*^2^ or the 2-D discrete space *Z*^2^. In the following, we refer to morphological operations defined on the discrete space. The flat erosion of *f* by *B* ⊂ *Z*^2^ is given by the following expression:

(1) $${ɛ}_{B}\left[f(\text{x},\text{y})\right]=\left(f\;\Theta B\right)\left(\text{x},\text{y}\right)=\min_{\left(\text{s},\text{t}\right)\in Z^{2}\left(B\right)}\left\{f\left(\text{x}+\text{s},\text{y}+\text{t}\right)\right\},\;\left(\text{x},\text{y}\right)\in Z^{2},$$

where *Z*^2^(B) denotes the set of discrete spatial coordinates associated to pixels lying within the neighborhood defined by a flat SE, designed by *B*. In this work, we will assume that the considered SE is symmetric with respect to its origin, i.e. *B* = *B̌*, where *B̌* denotes the reflection of *B* and is defined as *B̌* = {c:c = −b, for b ∈ *B*}. Similarly, the flat dilation of *f* by *B* is given by:

(2) $$\delta_{B}\left[f(\text{x},\text{y})\right]=\left(f⊕B\right)\left(\text{x},\text{y}\right)=\max_{\left(\text{s},\text{t}\right)\in {\text{Z}}^{2}\left(B\right)}\left\{f\left(\text{x}+\text{s},\;\text{y}+\text{t}\right)\right\},\;\left(\text{x},\text{y}\right)\in Z^{2},$$

In order to extend the above basic morphological operations to hyperspectral images, let us now consider a multi-channel image ***f***, defined on the N-D space. We impose an ordering relation in terms of spectral purity in the set of pixel vectors lying within a flat SE, designed by *B*, by defining a cumulative distance D*_B_*[***f***(x,y)] between one particular pixel ***f***(x,y), where ***f***(x,y) denotes the N-D vector at discrete spatial coordinates (x,y)*∈Z*^2^, and all the pixel vectors in the spatial neighborhood given by *B* (*B*-neighborhood):

(3) $${\text{D}}_{B}\left[f(\text{x},\text{y})\right]=\sum_{\text{s}}\sum_{\text{t}}\text{Dist}\left[f(\text{x},\text{y}),\;f\left(\text{s},\text{t}\right)\right],\;\left(\text{x},\text{y}\right)\in Z^{2},$$

where Dist is a point-wise distance measure between two N-dimensional vectors. As a result, D*_B_*[***f***(x,y)] is given by the sum of Dist scores between ***f***(x,y) and every other pixel vector in the *B*-neighborhood. To be able to define the usual MM operators in a complete lattice framework, we need to be able to define a supremum and an infimum given an arbitrary set of vectors S = {***s***_1_, ***s***_2_,…, ***s***_n_}, where n is the number of vectors in the set. This can be achieved by computing D*_B_*(S) and selecting ***s***_p_ such that D*_B_*⌊***s***_p_⌋ is the minimum of that set, with 1 ≤ p ≤ n. In similar fashion, we can select ***s***_k_ such that D*_B_*[***s***_k_] is the maximum of that set, with 1 ≤ k ≤ n. Based on the simple definitions above, the flat extended erosion of ***f*** by *B* is based on the selection of the *B*-neighborhood pixel vector that produces the minimum value for D*_B_*:

(4) $${ɛ}_{B}\left[f(\text{x},\text{y})\right]=\left(f\Theta B\right)(\text{x},\text{y})=\left\{f(\text{x}+\text{s}\prime ,\text{y}+\text{t}\prime ),(\text{s}\prime ,\text{t}\prime )={arg min}_{(\text{s},\text{t})\in {\text{Z}}^{2}\left(B\right)}\left\{{\text{D}}_{B}\left[f(\text{x}+\text{s},\text{y}+\text{t})\right]\right\}\right\}$$

where the argmin operator selects the pixel vector which is most highly similar, according to the distance Dist, to all the other pixels in the in the *B*-neighborhood. On other hand, the flat extended dilation of ***f*** by *B* selects the *B*-neighborhood pixel vector that produces the maximum value for D*_B_*:

(5) $$\delta_{B}\left[f(\text{x},\text{y})\right]=\left(f⊕B\right)(\text{x},\text{y})=\left\{f(\text{x}+\text{s}\prime ,\text{y}+\text{t}\prime ),\;(\text{s}\prime ,\text{t}\prime )=\arg\max_{(\text{s},\text{t})\in {\text{Z}}^{2}\left(B\right)}\left\{{\text{D}}_{B}\left[f(\text{x}+\text{s},\text{y}+\text{t})\right]\right\}\right\}$$

where the argmax operator selects the pixel vector that is most highly different, according to Dist, to all the other pixels in the *B*-neighborhood. By means of the proposed ordering strategy in (3), the erosion and dilation operations defined in (4) and (5) are a pair of adjunct operators with all the right algebraic properties. It should be noted that the proposed extended operators are vector-preserving in the sense that no vector (spectral constituent) that is not present in the input data is generated as a result of the extension process. Also, it is important to emphasize that the arg operators are essential to achieve the above goal. In multi-channel morphology, the minimum (respectively, maximum) is the pixel vector that minimizes (respectively, maximizes) the value of D*_B_*. On other hand, the choice of Dist is a key topic in the resulting multi-channel ordering relation. In this work, we consider two widely used pseudo-distances which are well-suited to hyperspectral image analysis [4]: the spectral angle distance (SAD) and the spectral information divergence (SID). It should be noted that SAD is invariant in the multiplication of the input vectors by constants and, consequently, is invariant to unknown multiplicative scalings that may arise due to differences in illumination and sensor observation angle (all vectors are normalized to the unit sphere so that magnitudes do not matter). In contrast, SID is based on the concept of divergence, and measures the discrepancy of probabilistic behaviors between the two spectral signatures [13].

### 3.3. Processing Examples

**Figure 2.**
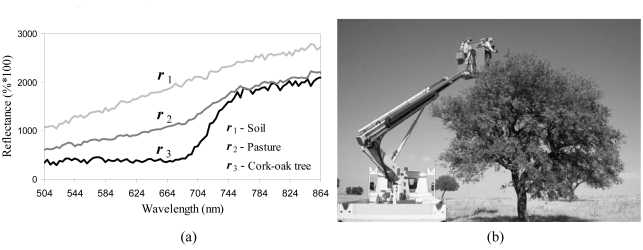
(a) ROSIS spectral signatures of soil (***r***_1_), pasture (***r***_2_) and cork-oak tree (***r***_3_). (b) Ground-truth data collection over a semi-arid test site using an ASD FieldSpec Pro spectro-radiometer.

**Figure 3.**
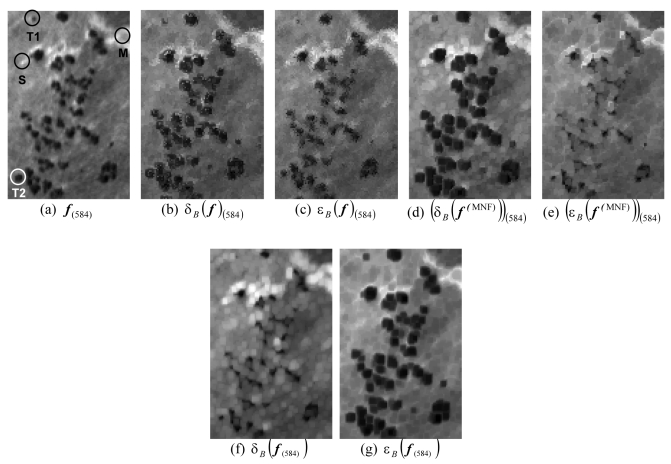
(a) Spectral band at 584 nm of a ROSIS hyperspectral image ***f***. (b) The same band in ***f*** ⊕ *B*. (c) The same band in ***f*** Θ *B*. (d) The same band in ***f***^(MNF)^ ⊕ *B*. (e) The same band in ***f***^(MNF)^ Θ *B*. (f) Monochannel dilation of (a). (g) Monochannel erosion of (a).

In order to illustrate the proposed approach, let *B* be a flat 3×3-pixel SE and let ***f*** be a hyperspectral scene, collected by the Reflective Optics Spectrographic Imaging System (ROSIS) sensor operated by German Aerospace Center (DLR) over a particular scenario: the so-called ‘Dehesa’ ecosystem, mainly formed by cork-oak trees, soil and pasture, in Caceres, SW Spain. Representative ROSIS spectral signatures of the three constituents above are displayed in Figure 2(a). The considered scene, displayed in Figure 3(a), consists of 88×134 pixels of 1.2 meters, each containing 92 spectral bands covering the spectral range from 504-864 nm. This scene has been selected for experiments due to its simplicity and the availability of ground-truth information, collected during a site visit to the area in July 2001. As part of our experiment, the data from this site visit were compiled as a spectral library of field measurements, obtained using an ASD FieldSpec Pro spectro-radiometer [see Figure 2(b)]. Figure 3(a) illustrates the spectral band collected at 584 nm wavelength by the ROSIS imaging spectrometer, denoted from now on as *f*_(584)_, where four representative pixels were identified and marked by circles: T1 (small cork-oak tree), T2 (medium-sized cork-oak tree), S (soil) and M (pasture), where most of the gray areas in the scene are actually formed by mixtures of soil and pasture. The four pixels above can be used as reference pixels in order to investigate the effect of different morphological operations.

The result of applying an extended erosion/dilation operation to ***f*** using *B* is a new data cube, with exactly the same dimensions as the original, where each pixel is replaced by the maximum/minimum of the neighborhood defined by the flat SE. Figures 3(b) and 3(c) show the 584 nm band of the resulting images after applying multi-channel dilation δ*_B_*(***f***), and erosion ε*_B_*(***f***), respectively (the pixel coordinates are removed for clarity). The resulting bands are denoted by δ*_B_*(***f***)_(584)_ and ε*_B_*(***f***)_(584)_. In both cases, SAD was used to define morphological transformations, and no specific strategy was applied to address multiple suprema (or infima) in the original feature space.

As shown by Figures 3(b) and 3(c), some border interferences can be observed in the edges of objects in the processed images as a result of the above situation. For comparative purposes, Figures 3(d) and 3(e) respectively show the same band in the resulting images after SAM-based multi-channel dilation δ*_B_*(***f***^(MNF)^) and erosion ε*_B_*(***f***^(MNF)^), respectively denoted by (δ*_B_*(***f***^(MNF)^))_(584)_ and (ε*_B_*(***f***^(MNF)^))_(584)_, where the superscript “(MNF)” indicates that this time the MNF-reduced feature space was used in conjunction with D-ordering to order those pixels that resulted in a tie in the original image. As can be seen in Figure 3(d), multi-channel dilation has the effect of expanding cork oak (dark) and soil (bright) areas, which mainly contain pure spectral signatures according to our ground experiments.

On the other hand, it can be seen in Figure 3(e) that multi-channel erosion expands gray-tone (“mixed”) areas and shrinks both dark (cork-oak) and bright (pure soil) areas. This time, no border intereferers were appreciated in the image objects (due to their similarity with MNF-based results, PCA-based results are not displayed in the example). Finally, Figures 3(f) and 3(g) respectively show the resulting images after applying monochannel erosion dilation δ*_B_*(***f***_(584)_) and erosion ε*_B_*(***f***_(584)_) to the band at 584 nm wavelength in Figure 3(a). As can be seen, monochannel dilation develops objects which appear as bright areas in the considered spectral channel, whereas monochannel erosion shrinks bright objects and develops dark areas in the same channel, regardless of the spectral purity of the samples.

## 4. Multi-Channel Morphological Profiles

As reported in previous work [3], there is a need for feature extraction methods that can reduce the dimensionality of the hyperspectral data to the right subspace without losing the crucial information that allows for the separation of classes. For that purpose, sequences of multi-channel morphological transformations with SE's of varying width will be used. The use of a range of different SE sizes to analyze the size distribution of structures in a scene is called granulometry [8]. In [22], a composition of mono-channel morphological operations based on SE's of different sizes in order to characterize image structures in high-resolution grayscale urban satellite data. The link between the morphological profiles and the spatial information (size and contrast of the features from the scene) is further explained in [23]. A simple technique to extend the above approaches to multi-channel imagery is to apply component transformations such as PCA or MNF, and then consider the first few components only as the baseline image for constructing morphological profiles based on mono-channel morphological filters, in band-by-band fashion [9]. However, our speculation in this work is that multi-channel MM filters should assist in creating a feature set which is more effective in the discrimination of image features. Below, we describe a framework for the construction of profiles based on multi-channel morphological operations.

The concept of morphological profile relies on opening and closing by reconstruction [8], a special class of morphological transformations that do not introduce discontinuities, and therefore preserve the shapes observed in input images. The basic contrast imposed by conventional opening and closing versus reconstruction-based opening and closing can be described as follows: conventional opening and closing remove the parts of the objects that are smaller than the SE, whereas opening and closing by reconstruction either completely removes the features or retains them as a whole. In order to define the concept of multi-channel morphological profiles using a simple notation, we have omitted the spatial coordinates of pixel vectors from the formulation for simplicity. It should be noted, however, that multi-channel morphological profiles defined below are calculated for each pixel vector in the input data. We first define the geodesic dilation operator $\delta_{B}^{\left(1\right)}$of under *g* as:

(6) $$\delta_{B}^{\left(1\right)}\left(f,g\right)=\max\left\{\delta_{B}\left(f\right),g\right\}$$

where ***f*** and ***g*** are two hyperspectral images and δ*_B_*(***f***) is the elementary multi-channel dilation [8]. Similarly, we define the geodesic erosion ${ɛ}_{B}^{\left(1\right)}$ as:

(7) $${ɛ}_{B}^{\left(1\right)}\left(f,g\right)=\min\left\{{ɛ}_{B}\left(f\right),g\right\}$$

where ε*_B_*(***f***) is the elementary multi-channel dilation. Then, successive geodesic dilations and erosions can respectively be obtained by:

(8) $$\delta_{B}^{\left(k\right)}\left(f,g\right)=\underset{k\text{times}}{\underset{︸}{\delta_{B}^{\left(1\right)}\left(\delta_{B}^{\left(1\right)}\left(\cdots \delta_{B}^{\left(1\right)}\left(f,g\right),g\right),g\right)}}\;{ɛ}_{B}^{\left(k\right)}\left(f,g\right)=\underset{k\text{times}}{\underset{︸}{{ɛ}_{B}^{\left(1\right)}\left({ɛ}_{B}^{\left(1\right)}\left(\cdots {ɛ}_{B}^{\left(1\right)}\left(f,g\right),g\right),g\right)}}$$

The reconstruction by dilation of ***f*** under ***g*** is then given by $\rho_{B}^{\delta }\left(f,g\right)=\delta_{B}^{\left(\infty \right)}\left(f,g\right)$, i.e., until idempotence is reached [8]. Similarly, the reconstruction by erosion of ***f*** under ***g*** is given by $\rho_{B}^{ɛ}\left(f,g\right)={ɛ}_{B}^{\left(\infty \right)}\left(f,g\right)$. With the above definitions in mind, the opening by reconstruction of size *k* of an image ***f*** can be simply defined as the reconstruction of ***f*** from the erosion of size *k* of ***f***:

(9) $$\gamma_{B}^{\left(k\right)}\left(f\right)=\rho_{B}^{\delta }\left({ɛ}_{B}^{\left(k\right)}\left(f\right),f\right)$$

and the closing by reconstruction is defined by duality:

(10) $$\varphi_{B}^{\left(k\right)}\left(f\right)=\rho_{B}^{ɛ}\left(\delta_{B}^{\left(k\right)}\left(f\right),f\right)$$

Using (9) and (10), multi-channel morphological profiles are defined as follows. The multi-channel opening profile is defined as the vector $p_{\text{i}}^{\gamma }\left(f\right)=\left\{\gamma_{B}^{\left(\text{i}\right)}\left(f\right)\right\}$, while the multi-channel closing profile is given by $p_{\text{i}}^{\varphi }\left(f\right)=\left\{\varphi_{B}^{\left(\text{i}\right)}\left(f\right)\right\}$, with i = {0, 1, …, *k*}. Here, $\varphi_{B}^{\left(\text{i}\right)}\left(f\right)=\gamma_{B}^{\left(\text{i}\right)}\left(f\right)=f$ by the definition of opening and closing by reconstruction [8]. We define the combined derivative profile Δ***p***_i_ as the vector:

(11) $$\Delta p_{\text{i}}=\left\{\text{SAD}\left[\gamma_{B}^{\left(\text{i}\right)}\left(f\right),\gamma_{B}^{\left(\text{i}-1\right)}\left(f\right)\right]\right\}\cup \left\{\text{SAD}\left[\phi_{B}^{\left(\text{i}\right)}\left(f\right),\phi_{B}^{\left(\text{i}-1\right)}\left(f\right)\right]\right\},\;\text{with}\;\text{i}=\left\{1,\text{ 2},\;\ldots ,\text{k}\right\}$$

**Figure 4.**
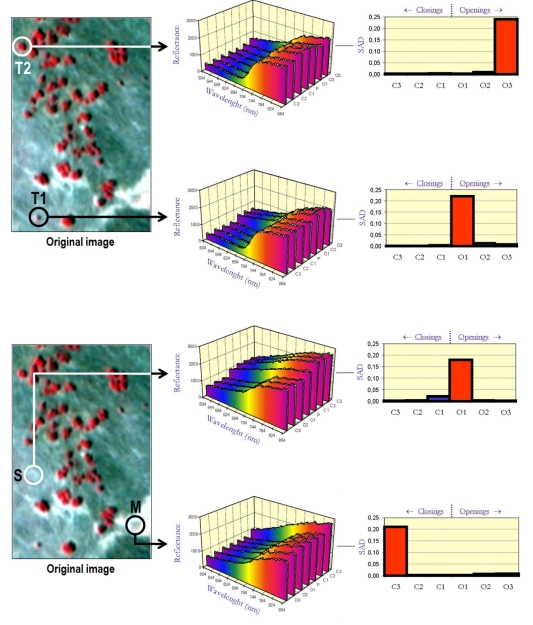
Construction of SAD-based multi-channel morphological profiles relative to a series of opening and closing iterations for several pixels in a ROSIS hyperspectral scene collected over a semi-arid area in Spain: T1 (pure tree of small size), T2 (pure tree of large size), S (pure soil), and M (mixed area formed by soil and pasture).

In order to illustrate the concept of multi-channel morphological profile, we use again the four target pixels in Figure 2(a). Ground-truth data collection revealed that, while T1, T2 and S can be considered spectrally pure at a macroscopic level, the ROSIS sensor spatial resolution was not large enough to separate soil from pasture at the pixel M. As a result, this pixel was labeled as spectrally mixed. Figure 4 illustrates the process for creating multi-channel morphological profiles for pixels T1, T2, S and M. In the figure, the ROSIS scene is shown using a false color composition and rotated with regards to Figure 2(a) for visualization purposes. As Figure 4 shows, pixels that are spectrally pure have a derivative profile that shows a high value in the opening series. In contrast, as can be seen for pixel M, mixed pixels have a derivative profile that shows the highest score in the closing series. The point where the derivative profile takes the maximum value can be used to record the most appropriate size of the SE for each pixel. As a result, the derivative profile can be used as a feature vector on which the classification is performed using a spatial/spectral criterion.

## 5. Experimental Results

### 5.1. Hyperspectral Image Data Sets

**Figure 5.**
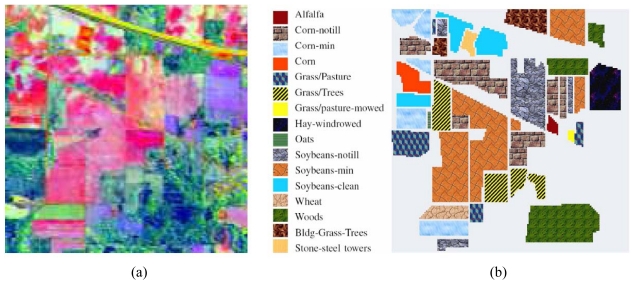
(a) False color composition of the AVIRIS Indian Pines scene. (b) Ground truth-map containing 16 mutually exclusive land-cover classes.

Three hyperspectral data sets have been used in our classification experiments. The first one was collected by the AVIRIS sensor over Northwestern Indiana in 1992. This scene, with a size of 145 lines by 145 samples, was acquired over a mixed agricultural/forest area, early in the growing season. The scene comprises 202 spectral channels (after elimination of water absorption and noisy bands) in the wavelength range from 0.4 to 2.5 μm, nominal spectral resolution of 10 nm, spatial resolution of 20 meters by pixel, and 16-bit radiometric resolution. After an initial screening, several spectral bands were removed from the data set due to noise and water absorption phenomena, leaving a total of 190 radiance channels to be used in the experiments. For illustrative purposes, Figure 5(a) shows a false color composition of the AVIRIS Indian Pines scene, while Figure 5(b) shows the ground-truth map available for the scene, displayed in the form of a class assignment for each labeled pixel, with 16 mutually exclusive ground-truth classes. These data, including ground-truth information, are available online from http://dynamo.ecn.purdue.edu/biehl/MultiSpec, a fact which has made this scene a widely used benchmark for testing the accuracy of hyperspectral data classification algorithms.

**Figure 6.**
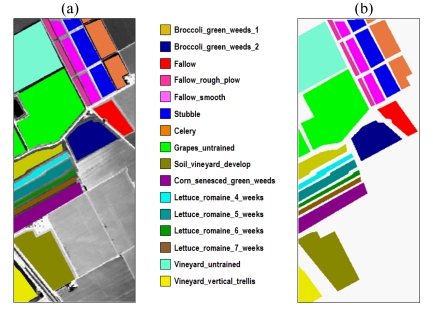
(a) Spectral band at 488 nm of an AVIRIS hyperspectral image comprising several agricultural fields in Salinas Valley, California, with ground-truth classes superimposed. (b) Ground-truth map containing 15 mutually exclusive land-cover classes.

The second AVIRIS data set used in experiments was collected over the Valley of Salinas in Southern California. The full scene consists of 512 lines by 217 samples with 190 spectral bands (after elimination of water absorption and noisy bands) from 0.4 to 2.5 μm, nominal spectral resolution of 10 nm, and 16-bit radiometric resolution. It was taken at low altitude with a pixel size of 3.7 meters. The data include vegetables, bare soils and vineyard fields. Figure 6(a) shows the entire scene and a sub-scene of the dataset (called hereinafter Salinas A), outlined by a white rectangle, which comprises 83 samples by 86 lines. Figure 6(b) shows the available ground-truth regions. As shown in Figure 6(b), ground-truth is available for about two thirds of the entire Salinas scene.

**Figure 7.**
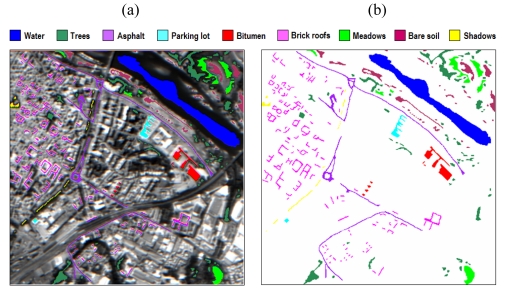
(a) Spectral band at 639 nm of a DAIS 7915 hyperspectral scene comprising urban features in Pavia, Italy, with ground-truth classes superimposed. (b) Ground-truth map containing 9 mutually exclusive land-cover classes.

The third data set used in experiments was collected by the DAIS 7915 airborne imaging spectrometer of the German Remote Sensing Data Center (DLR) over the city of Pavia, Italy. The spatial resolution is of 5 meters per pixel, and the scene consists of 400 lines by 400 samples. Figure 7(a) shows the spectral band collected at 639 nm, which reveals a dense residential area on one side of the river, as well as open areas and meadows on the other side. Ground truth information is available for several areas of the scene as shown in Figure 7(b), comprising the following land-cover classes: 1) water; 2) trees; 3) asphalt; 4) parking lot; 5) bitumen; 6) brick roofs; 7) meadows; 8) bare soil; 9) shadows. Following previous research studies on this scene [12, 13], we take into account only 40 spectral bands of reflective energy, and thus skip thermal infrared bands and middle infrared bands above 1958 nm because of low SNR in those bands.

### 5.2. Support Vector Machine Classification System

Before describing the results obtained in experimental validation, we first briefly describe the adopted supervised classification system. Firstly, relevant features for classification are extracted from the original image by using multi-channel morphological profiles constructed for labeled pixels according to the ground-truth. The resulting features were used to train an SVM classifier [6, 24] in which three different types of kernels: polynomial, Gaussian, and SAD-based were used. Kernel methods have shown success in hyperspectral imaging problems [25-27]. Specifically, the SVM was trained with each of these training subsets and then evaluated with the remaining test set. The use of dimension reduction techniques, known to affect hyperspectral data analysis [28-29], is also explored in our experiments. Each experiment was repeated five times, and the mean accuracy values were reported. Kernel parameters were optimized in all experiments by a grid search procedure [25]. In essence, the SVM classification is based on the notion of fitting an optimal separating hyperplane between classes by focusing on the training samples that lie at the edge of the class distributions, the support vectors [6]. All of the other training samples are effectively discarded as they do not contribute to the estimation of hyperplane location. In this way not only is an optimal hyperplane fitted, in the sense that it is expected to have a large degree of generalizability, but also a high accuracy may be obtained with the use of a small training set.

### 5.3. Experimental Design and Classification Results Using Hyperspectral Data

In this section, we provide experimental results using the two AVIRIS data sets described in subsection 5.1. The classification system described in subsection 5.2 is trained with different types of input features in supervised fashion. The five types of input features considered in the classification experiments conducted in this work can be summarized as follows:

1. **Original**. In this case, we use the (full) original spectral signatures available in the hyperspectral data as input to the proposed classification system. The dimensionality of the input features used for classification equals *N*, the number of spectral bands in the original data set.
2. **Reduced**. Here, we apply a dimensionality transformation (such as the MNF or the PCA) to the original input data so that the dimensionality of the input data is reduced and information is packed in the first components resulting after the transformation. In this case, we use the virtual dimensionality (VD) concept in [30] to estimate the dimensionality of the hyperspectral data set and then retain the first *p* components of the data after the dimensional transformation. As a result, the dimensionality of the input features used for classification in this particular case is *p*.
3. **Multi-channel**. In this case, we use multi-channel morphological profiles (with *k* opening/closing iterations) applied (in vector-based fashion) to the full spectral information available in the hyperspectral data. Here, the dimensionality of the input features (morphological profiles) used for classification is 2*k* (see Figure 4). Three types of vector ordering (L-ordering, D-ordering and R-ordering) are investigated in the construction of multi-channel profiles.
4. **Multi-channel reduced**. Here, we use multi-channel morphological profiles (with *k* opening/closing iterations) but applied (in vector-based fashion) to the first *p* components of the data resulting after applying a dimensionality transformation (either by PCA or MNF) to the original input data. The dimensionality of the input features used for classification is also 2*k* and three types of vector ordering (L-ordering, D-ordering and R-ordering) are investigated in the construction of the multi-channel reduced profiles.
5. **Mono-channel**. Finally, we also use mono-channel morphological profiles (with *k* opening/closing iterations) applied to the first component resulting from the PCA and MNF transformations. The dimensionality of the input features used for classification is also 2*k*.

It should be noted that the main difference between the last three types of input features (multi-channel, multi-channel reduced and mono-channel) is the amount of spectral information used to construct the morphological profiles, which goes from the full original spectral information to the first component after applying the PCA or MNF transform, but in all cases 2*k*-dimensional features are used as an input to the proposed SVM classifier.

#### 5.3.1. Experiment 1: AVIRIS Indian Pines Data Set

In this first experiment, we use the AVIRIS Indian Pines data set in Figure 5(a) to analyze the impact of the training set size in the proposed classification system. In order to validate the classification accuracy in several analysis scenarios using limited training samples, we resort to ground-truth measurements in Figure 5(b). Small training sets, composed of 2%, 4%, 6%, 8%, 10% and 20% of the ground-truth pixels available per class, are randomly extracted from the labeled pixels in Figure 5(b). Then, the five types of input features mentioned at the beginning of section 5.3.1 were constructed for the selected training samples. The dimensionality of the input data, as estimated by the VD concept, was *p*=16. In order to construct the morphological profiles, the number of opening/closing iterations was empirically set to *k* = 10 after testing different values for this parameter (the impact of this parameter will be thoroughly evaluated in the following experiment).

Table 1 summarizes the overall classification accuracies obtained by the SVM classifier using the three considered kernels. For the multi-channel profiles, Table 1 reports the results obtained by D-ordering, L-ordering and R-ordering. From Table 1, it can be seen that SVMs generalize quite well: with only 2% of training pixels per class, about 85% overall classification accuracy is reached by all kernels (more than 91% for the Gaussian kernel) when trained using multi-channel profiles and their reduced versions. In all cases, classification accuracies decreased when mono-channel profiles or the original spectral information (even after dimensionality reduction) were used for the training stage. This confirms the fact that SVMs are less affected by the Hughes phenomenon, in particular, when trained with feature vectors obtained using spatial and spectral information. It is also clear from Table 1 that the classification accuracy is generally correlated with the training set size. However, when multi-channel morphological profiles were used to construct the feature vectors, higher classification accuracies were achieved with less training samples. Finally, the classification results based on the original spectral information required a higher number of training samples to achieve comparable results due to the high dimensionality of the input feature vectors.

The results reported in Table 1 indicate the importance of including both spatial and spectral information in the SVM classifier. The proposed approach for multi-channel morphological feature extraction seems more effective than mono-channel morphological profiles for combining such spatial and spectral information in the extraction of relevant features for SVM-classification, in particular, when a D-ordering strategy was used, which outperformed both R-ordering and L-ordering in this experiment. Finally, it can be seen in Table 1 that the best classification scores were generally achieved for the Gaussian kernel, in which the overall accuracy obtained with 2% of the training pixels per class is only 2.59% lower than the overall accuracy obtained with 20% of the training pixels per class (extracted using multi-channel morphological profiles with D-ordering). On the other hand, the SAM-based kernel gives slightly degraded classification results. However, with accuracies above 85% in a challenging classification problem, this kernel also provides quite promising results. Finally, the polynomial kernel needs more training samples than the two other kernels to perform appropriately, as can be seen from the lower classification accuracies obtained by this kernel for a limited number of training samples.

#### 5.3.2. Experiment 2: AVIRIS Salinas Data Set

In this second experiment, we used the AVIRIS Salinas data set in Figure 6(a) to analyze the impact of the number of opening/closing iterations in the construction of mono-channel and multi-channel morphological profiles for training the proposed classification system. As in the previous experiment, the other parameter to be investigated in this experiment is the type of vector ordering strategy used to construct the multi-channel morphological profiles. For that purpose, a random sample of only 2% of the pixels was chosen from the known ground-truth of the fifteen ground-truth classes in Figure 6(b). Then, the five types of input features mentioned at the beginning of Section 5.3.1 were constructed for the selected training samples. The dimensionality of the input data, as estimated by the VD concept, was *p*=22. In order to construct the multi-channel morphological profiles, three types of vector ordering (L-ordering, D-ordering and R-ordering) were used. The trained classifier was then applied to the remaining 98% of the known ground pixels in the scene. In all cases, an SVM classifier with Gaussian kernel was used to produce the final classification scores.

**Figure 8.**
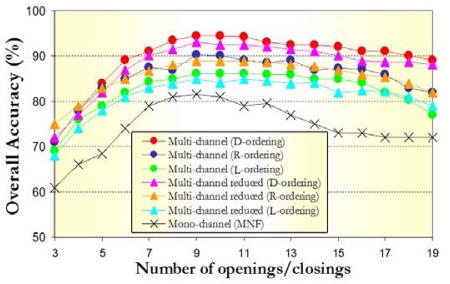
Overall test classification accuracies obtained after applying the proposed SVM-based classification system (with Gaussian kernel) to multi-channel and mono-channel morphological profiles (with different numbers of opening/closing iterations) built for the AVIRIS Salinas data set.

Figure 8 displays the overall test classification accuracies obtained after applying our classification system to multi-channel and mono-channel morphological profiles as a function of the number of opening/closing operations. Three different approaches were tested in the construction of multi-channel morphological operations (L-ordering, D-ordering and R-ordering). Similarly, two different approaches were considered in the construction of mono-channel and multi-channel reduced profiles based on processing the first MNF components (PCA resulted in slightly lower classification accuracies and results based on this transform are omitted in this experiment for space considerations). As demonstrated by Figure 8, the best overall accuracies were achieved when multi-channel morphological profiles based on D-ordering were used for feature extraction. It should also be noted that R-ordering performed better than L-ordering when constructing such profiles. This fact revealed that D-ordering and R-ordering schemes are more appropriate than L-ordering for this application. Interestingly, very similar classification scores were obtained by multi-channel reduced morphological profiles based on processing the first *p* = 22 components resulting from the MNF transform instead of the full spectral information. In this case, again D-ordering and R-ordering resulted in better classification results than L-ordering. Finally, it is clear from Figure 8 that the results produced by multi-channel and multi-channel reduced features were superior than those found using mono-channel features. From Figure 8 it is also apparent that the width in pixels of classes of interest in the Salinas AVIRIS scene makes *k* = 9 opening/closing iterations a reasonable parameter selection for most of the methods tested in this experiment. The construction of morphological feature vectors with larger data dimensions generally causes a loss in the classification performance.

To conclude this experiment, Table 2 reports the overall and individual test classification accuracies for each of the classes in the Salinas data set, using *k* = 9 opening/closing iterations for the construction of multi-channel and mono-channel morphological profiles prior to SVM-based classification. The results obtained by using the original spectral information in the hyperspectral scene are also shown for comparison. As can be examined in Table 2, the classification accuracies obtained after using multi-channel morphological profiles based on D-ordering and R-ordering are higher than the accuracies obtained after using multi-channel morphological profiles based on L-ordering. This confirms the effectiveness of D-ordering and R-ordering with regards to L-ordering in this example. Comparison between D-ordering and R-ordering also points out that the use of D-ordering ordering leads to slightly better results. It is also clear from Table 2 that the proposed multi-channel morphological profiles provide feature vectors which are more useful than their mono-channel counterparts in terms of classification accuracies.

Interestingly enough, however, a deeper analysis of the results in Table 2 reveals some limitations in the proposed techniques. For example, the individual test accuracies obtained after using multi-channel morphological profiles based on D-ordering and R-ordering on the *Broccoli_green_weeds_1*, *Corn_senesced_weeds* and four *Lettuce_romaine* (at different weeks since planting) classes are only slightly better than those found after using mono-channel morphological profiles or the original spectral information for SVM-based classification. It should be noted that the above six classes are dominated by directional features. As a result, the use of directional SE's (instead of disks of increasing size) in the construction of morphological profiles may assist in better characterizing those features, in particular, in more complex analysis scenarios such as urban environments, typically characterized by nested regions. Further experiments using hyperspectral data sets collected over urban areas are highly desirable.

#### 5.3.3. Experiment 3: DAIS 7915 Data Set Over Pavia, Italy

In this third experiment, we use the DAIS 7915 urban data set in Figure 7(a) to analyze the performance of the proposed techniques in a challenging urban data analysis scenario. In this experiment, a maximum in the overall classification accuracy reported for the proposed multi-channel morphological profiles was generally observed when the number of opening/closing operations was set to 10. The main aspect to be investigated in this experiment is the type of vector ordering strategy used to construct such multi-channel morphological profiles. For that purpose, a random sample of only 2% of the pixels was chosen from the known ground-truth of the nine ground-truth classes in Figure 7(b). Then, the five types of input features mentioned at the beginning of section 5.3.1 were constructed for the selected training samples. The dimensionality of the DAIS 7915 data, as estimated by the VD concept, was *p* = 15. In order to construct the multi-channel morphological profiles, three types of vector ordering (L-ordering, D-ordering and R-ordering) were used. The trained classifier was then applied to the remaining 98% of the known ground pixels in the scene. In all cases, an SVM classifier with Gaussian kernel was used to produce the final classification scores.

As shown by Table 3, the classification accuracies obtained after using multi-channel morphological profiles based on D-ordering are higher than those obtained after using multi-channel morphological profiles based on R-ordering and L-ordering, in particular, for complex urban classes with nested regions such as *Brick roofs, Asphalt*, or *Shadows*. Comparison between D-ordering and R-ordering also points out that the use of D-ordering ordering leads to better characterization of spatially homogeneous classes, such as *Bare soil, Meadows* or *Water*. It is also clear from Table 3 that the proposed multi-channel morphological profiles (based on D-ordering and R-ordering) provide feature vectors which can be more useful than their mono-channel counterparts in terms of individual and overall classification accuracies. Finally, multi-channel reduced profiles provide results which are close to those obtained by the multi-channel profiles in this experiment.

As a final major comment, we should remark that results reported in the three reported experiments were obtained by using SAD as the base distance for the construction of multi-channel morphological operations. The same experiments were also conducted using the SID distance, with very similar results (omitted here for space considerations.)

## 6. Conclusions and Future Research

In this paper, we have addressed the problem of supervised classification of hyperspectral image data with limited training samples and further investigated several strategies to build morphological profiles by considering the full spectral information available in the input hyperspectral data and different ways to reduce its dimensionality. We have also given special attention to the issue of how to order hyperspectral pixel vectors in order to define morphological operations by extension when considering multiple spectral channels. Our experimental results, conducted using three highly representative data sets collected by the AVIRIS and DAIS 7915 sensors, reveal that multi-channel morphological profiles built using the entire spectral information available in the data can provide a very good mechanism for feature extraction prior to classification by integrating the spatial and the spectral information available in the data. However, an important aspect revealed by experiments is that the vector ordering adopted when constructing such profiles has an important impact on the final outcome and therefore has to be carefully selected (in our case, a spectral distance-based ordering provided better results than other strategies tested such as conditional ordering). Since the construction of multi-channel morphological profiles using the full spectral information can be computationally expensive, we have also provided a mechanism to build the profiles on the data resulting from a dimensional transformation such as the PCA or the MNF (assisted by the VD concept to automatically select the dimensionality of the reduced feature space).

A potential drawback in the proposed morphological approaches has to do with the need to heed a range of morphological filters with increasing SE sizes, a labor which results in a heavy computational burden when processing hyperspectral data. This phenomenon is particularly relevant for the case of images with large and spectrally homogeneous regions. However, it has been shown in previous work that morphological operations for hyperspectral image analysis can be effectively implemented in parallel [31]. Further research should comprise more intelligent approaches to construct the training sets on which morphological profiles are built to train the proposed SVM classifier as well as the study of alternative approaches to be used in the extension of morphological operations to high dimensional spaces. Comparisons of the proposed approach to other recent works such as the technique described in [32], which performs concatenation of extended morphological profiles and spectral information, both after feature extraction, and SVM classification (with a kernel), or the technique described in [33], which combines composite kernels and SVM classification, are also worthy of exploration in future work.

## Figures and Tables

**Table 1.** Overall classification accuracies (in percentage) obtained after applying the proposed SVM classification system (with polynomial, Gaussian and SAM-based kernels) to mono-channel and multi-channel morphological profiles built for the Salinas AVIRIS scene with 190 spectral bands. Results using the original spectral information and a reduced version of the original scene (obtained after applying the MNF transform and retaining the first *p* = 16 components) are also displayed.

**Training set size**		**2%**	**4%**	**6%**	**8%**	**10%**	**20%**
Polynomial kernel	Original	79.45	79.88	81.15	81.39	82.49	83.01
	Reduced (MNF)	82.33	82.94	83.21	83.82	85.34	86.12
	Multi-channel (D-ordering)	**85.06**	**85.93**	**86.78**	**87.24**	**88.03**	**88.97**
	Multi-channel (L-ordering)	80.56	80.89	81.12	81.23	81.57	82.26
	Multi-channel (R-ordering)	82.95	83.89	84.12	85.40	86.17	87.15
	Multi-channel reduced (MNF)	84.21	84.42	84.90	86.21	87.48	89.95
	Mono-channel (MNF)	78.01	78.69	79.30	80.22	81.50	81.98

Gaussian kernel	Original	81.25	82.03	83.33	83.78	84.17	85.69
	Reduced (MNF)	87.94	88.23	88.78	88.96	89.45	89.48
	Multi-channel (D-ordering)	**91.44**	**92.45**	**92.68**	**92.97**	**93.25**	**94.03**
	Multi-channel (L-ordering)	80.67	81.78	81.56	81.90	82.03	82.16
	Multi-channel (R-ordering)	89.57	90.19	90.68	90.93	91.48	92.03
	Multi-channel reduced (MNF)	90.22	91.21	92.06	92.88	93.14	93.77
	Mono-channel (MNF)	80.45	80.59	80.98	81.16	81.29	82.06

SAM-based kernel	Original	84.25	84.89	85.33	85.90	86.45	87.22
	Reduced (MNF)	85.90	86.22	86.49	87.03	87.56	88.09
	Multi-channel (D-ordering)	**88.78**	**89.56**	**90.43**	**90.91**	**91.56**	**92.35**
	Multi-channel (L-ordering)	81.08	81.49	82.18	82.57	82.93	83.24
	Multi-channel (R-ordering)	86.17	86.94	87.35	87.80	88.54	89.12
	Multi-channel reduced (MNF)	86.83	87.55	88.25	89.33	90.42	90.69
	Mono-channel (MNF)	81.05	82.17	82.46	82.98	83.46	83.90

**Table 2.** Overall and individual test classification accuracies (in percentage) obtained after applying the proposed SVM classification system (with Gaussian kernel), using mono-channel and multi-channel morphological profiles with *k* = 9 opening/closing operations, to the Salinas AVIRIS scene with 190 spectral bands. Results using the original spectral information and an MNF-based reduced version of the original scene (obtained after retaining the first *p* = 22 components) are also displayed.

**Class**	Original	Reduced (MNF)	Multi-channel (D-ordering)	Multi-channel (R-ordering)	Multi-channel (L-ordering)	Multi-channel reduced (D-ordering)	Multi-channel reduced (R-ordering)	Multi-channel reduced (L-ordering)	Mono-channel (MNF)
Broccoli_green_weeds_1	78.42	76.25	82.64	79.36	77.33	81.25	79.01	77.89	76.21
Broccoli_green_weeds_2	80.13	79.45	86.31	81.26	80.28	83.02	81.17	79.31	74.58
Fallow	92.98	91.03	98.15	97.54	93.21	96.59	95.40	92.02	88.51
Fallow_rough_plow	96.51	94.23	96.51	95.30	91.90	94.52	92.37	89.43	86.77
Fallow_smooth	93.72	90.49	97.63	95.89	93.21	95.01	92.89	89.12	89.35
Stubble	94.71	91.55	98.96	95.48	95.43	98.02	95.17	91.24	85.19
Celery	89.34	86.01	98.03	96.75	94.28	99.05	93.67	93.23	88.40
Grapes_untrained	88.02	85.67	95.34	92.31	86.38	93.78	90.67	83.98	83.07
Soil_vineyard_develop	88.55	89.32	90.45	87.32	84.21	89.13	88.34	82.90	78.13
Corn_senesced_weeds	87.46	88.05	82.54	80.46	75.33	83.90	84.02	74.71	70.28
Lettuce_romaine_4_wk	78.86	77.23	83.21	81.42	76.34	82.28	81.49	77.82	73.10
Lettuce_romaine_5_wk	91.35	90.07	82.14	77.43	77.80	79.28	78.09	76.43	72.57
Lettuce_romaine_6_wk	88.53	86.54	84.56	80.76	78.03	81.81	79.15	78.19	74.25
Lettuce_romaine_7_wk	84.85	83.21	86.57	84.76	81.54	84.23	81.47	80.00	76.21
Vineyard_untrained	87.14	84.19	92.93	89.23	84.63	91.27	87.81	84.19	80.04

**Overall accuracy**	87.25	86.03	94.82	90.45	86.21	93.12	89.03	85.02	81.43

**Table 3.** Overall and individual test classification accuracies (in percentage) obtained after applying the proposed SVM classification system (with Gaussian kernel), using mono-channel and multi-channel morphological profiles with *k* = 10 opening/closing operations, to the DAIS 7915 scene over the city of Pavia with 40 spectral bands. Results using the original spectral information and an MNF-based reduced version of the original scene (obtained after retaining the first *p* = 15 components) are also displayed.

**Class**	Original	Reduced (MNF)	Multi-channel (D-ordering)	Multi-channel (R-ordering)	Multi-channel (L-ordering)	Multi-channel reduced (D-ordering)	Multi-channel reduced (R-ordering)	Multi-channel reduced (L-ordering)	Mono-channel (MNF)
Water	94.03	93.58	97.69	94.12	90.26	95.88	93.12	90.10	92.97
Trees	92.55	93.29	96.20	93.45	89.34	95.02	92.28	88.67	91.65
Asphalt	91.40	90.46	95.26	91.23	87.55	94.89	90.44	85.44	90.27
Parking lot	89.36	88.45	93.12	90.45	84.21	92.26	88.63	87.56	89.56
Bitumen	88.07	87.12	91.49	90.67	83.67	91.67	89.05	89.17	88.23
Brick roofs	84.93	85.79	93.28	89.23	82.45	92.65	89.12	89.45	90.40
Meadows	87.97	89.90	92.56	89.02	86.49	91.21	88.77	84.28	87.59
Bare soil	87.52	87.46	94.45	90.47	88.36	93.26	89.90	85.33	89.56
Shadows	89.26	89.56	95.89	92.98	88.03	94.92	91.46	84.91	90.23

**Overall accuracy**	89.46	88.43	94.91	92.06	87.15	93.89	91.27	86.94	89.17
